# Carry-over effect of learned distractor suppression on visual working memory

**DOI:** 10.3389/fnins.2026.1768751

**Published:** 2026-03-19

**Authors:** Nithin George, Pal C. Patel

**Affiliations:** School of Arts and Sciences, Ahmedabad University, Navrangpura, India

**Keywords:** attention, perception, selection history, visual statistical learning, working memory

## Abstract

Recent research has demonstrated that visual statistical learning guides attentional priority by proactively suppressing locations where distractors are more frequent. While there is evidence to suggest that both attention and working memory (WM) consume a common pool of cognitive resources, there is limited evidence on the nature of the interaction between the learned distractor suppression and WM. The present study investigated whether the learned spatial suppression of an external distractor has a carry-over effect that impairs the WM representation of objects encoded at that location. Across two experiments, participants first completed a visual search task designed to induce statistical learning. A salient singleton distractor appeared more frequently in a specific quadrant, while appearing less frequently in other locations. This was followed by a delayed discrimination task, in which participants had to encode and maintain target shapes presented at either the high-probability or low-probability distractor location. Experiment 1 imposed a low WM load (one target), while Experiment 2 imposed a high WM load (two targets). The results showed that the carry-over effect of suppression from a visual search task to a WM task was contingent on WM load. When the load was high, participants exhibited significantly reduced sensitivity (d’) and a higher false alarm rate for targets presented at the high-probability distractor location. These findings suggest that statistical learning operates by down-regulating sensory gain at suppressed locations. This pattern supports a non-modular, shared resource account of attention and WM, suggesting that history-driven attentional biases can modulate the WM representations.

## Introduction

The overwhelming array of sensory input is organized for perceptual inference through the selective processing by the attentional system. Classical models of attention attribute this selective guidance to top-down goals and bottom-up salience (Corbetta and Shulman, 2002; Desimone and Duncan, 1995). However, recent proposals have theorized attention beyond the bottom-up/top-down dichotomy, emphasizing the role of statistical learning of environmental regularities in shaping attentional priority. According to this tripartite view, observers continuously extract probabilistic information from the environment to both select the task-relevant stimulus and suppress the irrelevant distractors (Fiser and Aslin, 2001; Turk-Browne et al., 2005). The lingering biases in the attentional priority map selectively facilitate frequently co-occurring targets (Chun and Jiang, 1998) and suppress high probability distractor locations (Chelazzi et al., 2014; Ferrante et al., 2018; Wang and Theeuwes, 2018). This effect is observed when regularity is associated with locations, features (Goschy et al., 2014; Failing and Theeuwes, 2018; Stilwell et al., 2019), and time (Zhao et al., 2013).

### Visual statistical learning in attention

Visual statistical learning (VSL) enables the extraction of environmental regularities to guide attentional modulation, largely without conscious awareness. In visual search tasks, a salient distractor typically captures attention and slows down the target identification response time (RT) (Theeuwes, 1991, 1992). For instance, if the task is to find a diamond-shaped target in an array of circles, the odd-colored circle (singleton) captures attention, even though its color is a task-irrelevant dimension. Wang and Theeuwes (2018) used an adapted version of this additional singleton task to test whether repeating the singleton distractor at a specific location could improve the target identification RT. This statistical regularity in the spatial distribution of the singleton distractor led to faster RTs in trials in which the singleton distractor was presented more often (high-probability distractor location; HPDL).

Numerous studies have reported this “selection history” effect, positing it as a consequence of the dynamic adaptation of attentional weights in the priority map (Failing and Theeuwes, 2018). Neuroimaging findings suggest that the HPDL is proactively suppressed in the visual cortex, and the suppression corresponds to the retinotopic location of the HPDL (Ferrante et al., 2018; Wang et al., 2019). The suppression of HPDL occurs even before stimulus onset and thus indicates preparatory suppression of HPDL, which also generalizes to conditions in which the target or a neutral object is presented at HPDL (but see Huang et al., 2025). The effect of VSL on perception is not pre-attentive (Duncan et al., 2025; Musz et al., 2015) and it leads to lasting changes in the attentional priority map (Lengyel et al., 2021), and adapts to changes in the regularity (Theeuwes et al., 2022). While this history-driven process guides external attention, it is not clear whether the externally directed suppression interacts with internal cognitive processes, such as visual WM.

### Working memory and attentional learning

To investigate the interaction between WM and attentional learning, it is essential to consider the architectural overlap between selective attention and WM. According to the *shared resource hypothesis*, selective attention and WM are non- modular and recruit a common pool of capacity-limited neural substrates (Awh and Jonides, 2001; Ma et al., 2014). Specifically, the fronto-parietal network has overlapping regions involved in selective attention and WM (Corbetta et al., 2002). As the overlap in neuroanatomical areas does not entail a functional overlap, studies must establish evidence for WM and attention interaction that is not epiphenomenal. Researchers have primarily focused on the question of whether WM and attention share the same capacity limitation (Cowan, 2001; Oberauer, 2019). Evidence from individual differences studies shows a strong positive correlation between WM capacity and the ability to control attention, suggesting that the maintenance of information and the filtering of distractors rely on a common underlying mechanism (Engle, 2002; Kane et al., 2001). Additionally, dual-task paradigms have consistently shown that increasing WM load significantly impairs performance on selective attention tasks, indicating that these domains compete for a finite pool of processing resources (de Fockert et al., 2001; Lavie et al., 2004).

In the context of VSL, the modular view is supported by two lines of evidence, suggesting a potential independence between history-driven attention and WM. Firstly, most experiments investigating the effect of VSL on attention reported that the participants were unaware of the regularity (Musz et al., 2015; Xu et al., 2023), supporting the view that the adaptation of the attentional priority map through VSL is automatic and unconscious (Theeuwes et al., 2022; Wang and Theeuwes, 2018). Thus, VSL operates in a habit-like, automatic process that is independent of WM (Gao and Theeuwes, 2020b). Secondly, studies have shown that learning to suppress HPDL is unimpeded in the presence of a concurrent task that places a high cognitive load during learning, pointing to an independent mechanism that is not mediated by explicit top-down control (Gao and Theeuwes, 2020a; Gaspar et al., 2016; but see de Waard et al., 2023).

In contrast, there is also evidence suggesting an interactive effect of WM on attentional learning (Chidharom and Carlisle, 2024). For example, individual differences in WM capacity influence the VSL ability, implying that statistical learning draws WM resources (McCarter, 2021). WM capacity, measured by the number of to-be-remembered items, is reduced when items have a “selection-history,” especially when the WM load is high (Kuo, 2016). This suggests that the lingering effect of past attentional selection leads to proactive interference on the WM task. Studies have reported that statistical regularities affect WM by increasing its efficiency (Kaiser et al., 2015; Brady et al., 2009). However, there is little evidence for a carry-over effect of VSL-induced distractor suppression across consecutive WM tasks.

Despite the clear mechanistic overlap between attention and WM, empirical evidence regarding the impact of selection history on WM remains conflicting. The view, characterizing history-driven attention as a habit-like, automatic process that operates independently of WM resources (Gao and Theeuwes, 2020b), predicts that learned suppression should not tax WM capacity. Conversely, the shared resource view, supported by evidence that VSL is sensitive to WM availability (Chidharom and Carlisle, 2024; Kuo, 2016), predicts that selection history can modulate WM capacity. Existing studies have largely focused on how WM load affects the learning of regularities, or how history facilitates WM (Kaiser et al., 2015). There is a lack of evidence regarding the lingering effects of selection history on WM. Specifically, whether the proactive suppression of a location creates a “representational blind spot” that impairs the quality of WM items encoded at that location.

### Present study

If visual statistical learning operates by down-regulating neural excitability or synaptic gain at specific retinotopic locations to facilitate distractor rejection (Ferrante et al., 2018; Theeuwes et al., 2022), this suppression should degrade the sensory quality of any information processed at that location. Consequently, the external suppression of a location during visual search might translate into an internal deficit when that same location is recruited for WM task, effectively creating a representational blind spot (Awh and Jonides, 2001; Mayer et al., 2007; Postle, 2006). To empirically test this, the present study merged a visual search task with biased distractor location probability (Wang and Theeuwes, 2018; Theeuwes et al., 2022) and a WM task (Mayer et al., 2007). The location of the distractor was repeated at a specific location. Based on prior reports, this probabilistic bias will lead to visual statistical learning and consequently reduce the distractor interference on search RT (Ferrante et al., 2018; Wang and Theeuwes, 2018). The visual search task was followed by a delayed discrimination task, where we assessed whether the learned suppression of a location creates a carry-over effect on WM task performance. We hypothesized that if VSL reshapes the shared spatial priority map, then targets to be encoded onto WM appearing at the previously suppressed HPDL would suffer (Awh and Jonides, 2001; Postle, 2006; Ma et al., 2014). Furthermore, we predicted that this deficit would be most pronounced under high WM load, where the executive resources that might otherwise compensate for degraded sensory input are fully depleted (Fougnie and Marois, 2006; Ma et al., 2014).

## Materials and methods

### Participants

A total of 43 undergraduate students participated in Experiment 1 (34 females, nine males, Mean Age = 19.62, SD = 1.099, with an age range of 18–23 years) and 45 students (45 females, Mean Age = 20.20, SD = 0.85, with an age range of 19–23 years) participated in Experiment 2. Data from four participants were excluded from the final analysis - two from Experiment 1 and two from Experiment 2 - due to lower accuracy (<60 %) in the main task. All the participants had normal or corrected-to-normal vision. All participants provided informed consent before the experiment, and the experiment protocol was approved by the Ethics Committee of Ahmedabad University. After completing the experiment, participants were given monetary compensation of INR 100.

### Apparatus, stimuli and task

The experiment was conducted in a dimly lit, sound-attenuated room. Participants were seated 60 cm from the computer screen, controlled by a Windows PC. The experiment was designed and run using custom code written in MATLAB 2023b with a PsychtoolBox Version 3 (Brainard, 1997; Pelli, 1997). The data collected was then transferred to Microsoft Excel. The data analysis was then performed in RStudio (Posit Team, 2025). The experiments consisted of two phases: a Visual Search phase (Additional Singleton Task) and a WM phase (Delayed Discrimination Task).

#### Visual search task (phase 1)

**FIGURE 1:**
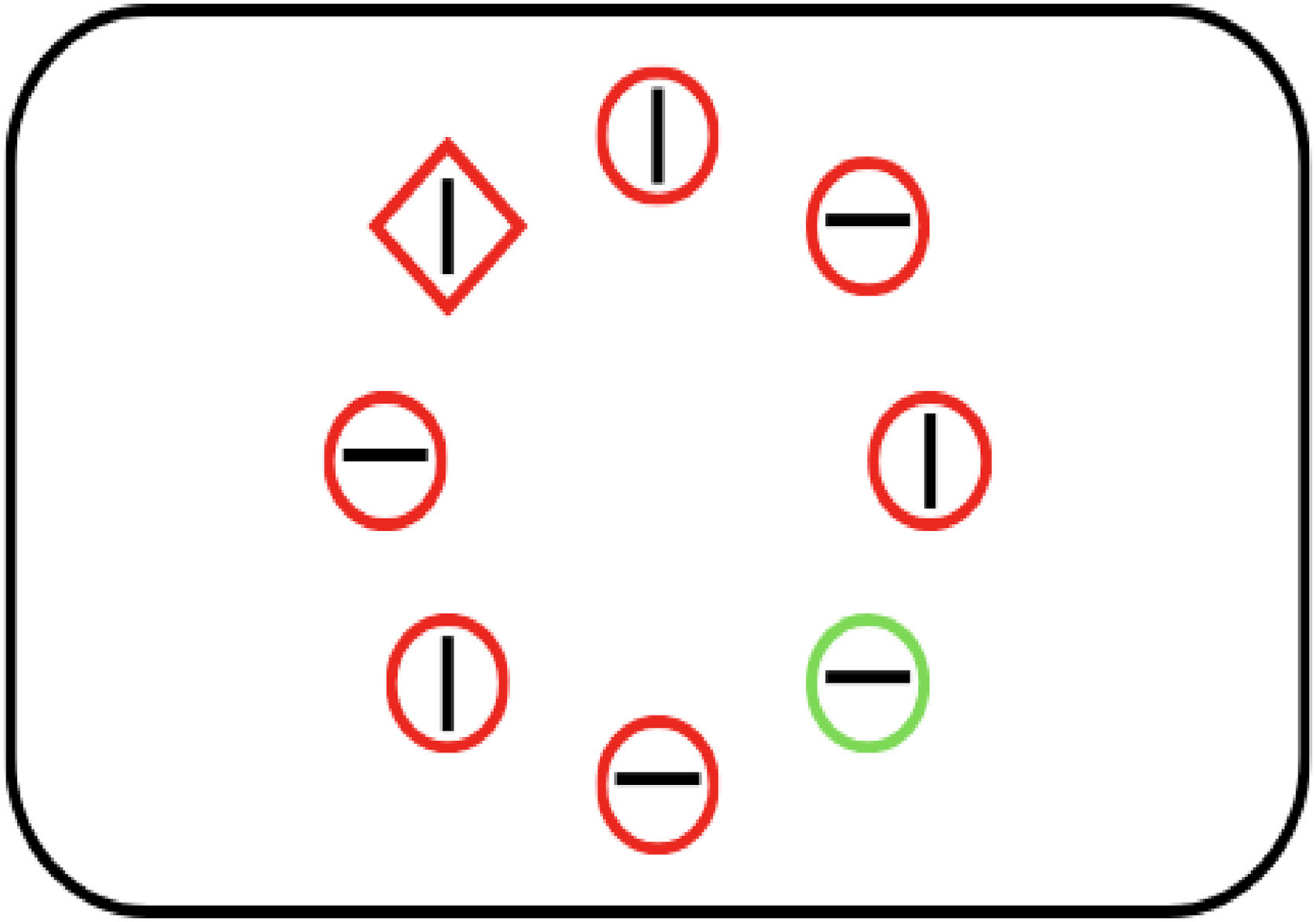
Visual search array with additional singleton for both Experiments 1 and 2. Participants searched for the object with the odd shape (red diamond), while ignoring the object with odd color (green circle). Participants reported the orientation of the line segment inside the odd shape (target).

In the first phase, participants performed a visual search task with an additional singleton (Figure 1). Eight geometrical shapes were presented in the perimeter of an imaginary circle, with a visual angle of 5.23°. The items were centered at the middle of the screen. Target and distractor items could appear at any of the eight equidistant positions around the imaginary circle. Of these eight items, one was defined as the target (an odd shape; circle as target, diamond as distractors, or vice-versa). Of the seven distractor items, one was an odd-colored distractor. The search items were circles and diamonds, which could be presented in either red (255, 0, 0) or green (0, 255, 0 color). In the singleton absent condition, all 8 items were presented in gray (128, 128, 128). The target shape/color and distractor color/shape were randomized across trials. In the singleton present condition, one of the distractors was presented in a color different from that of the target and the remaining distractors. A line segment was presented inside the geometrical shapes (target and distractors). The line segment had an equal probability of being oriented vertically or horizontally at every location.

#### Delayed discrimination task (phase 2)

**FIGURE 2:**
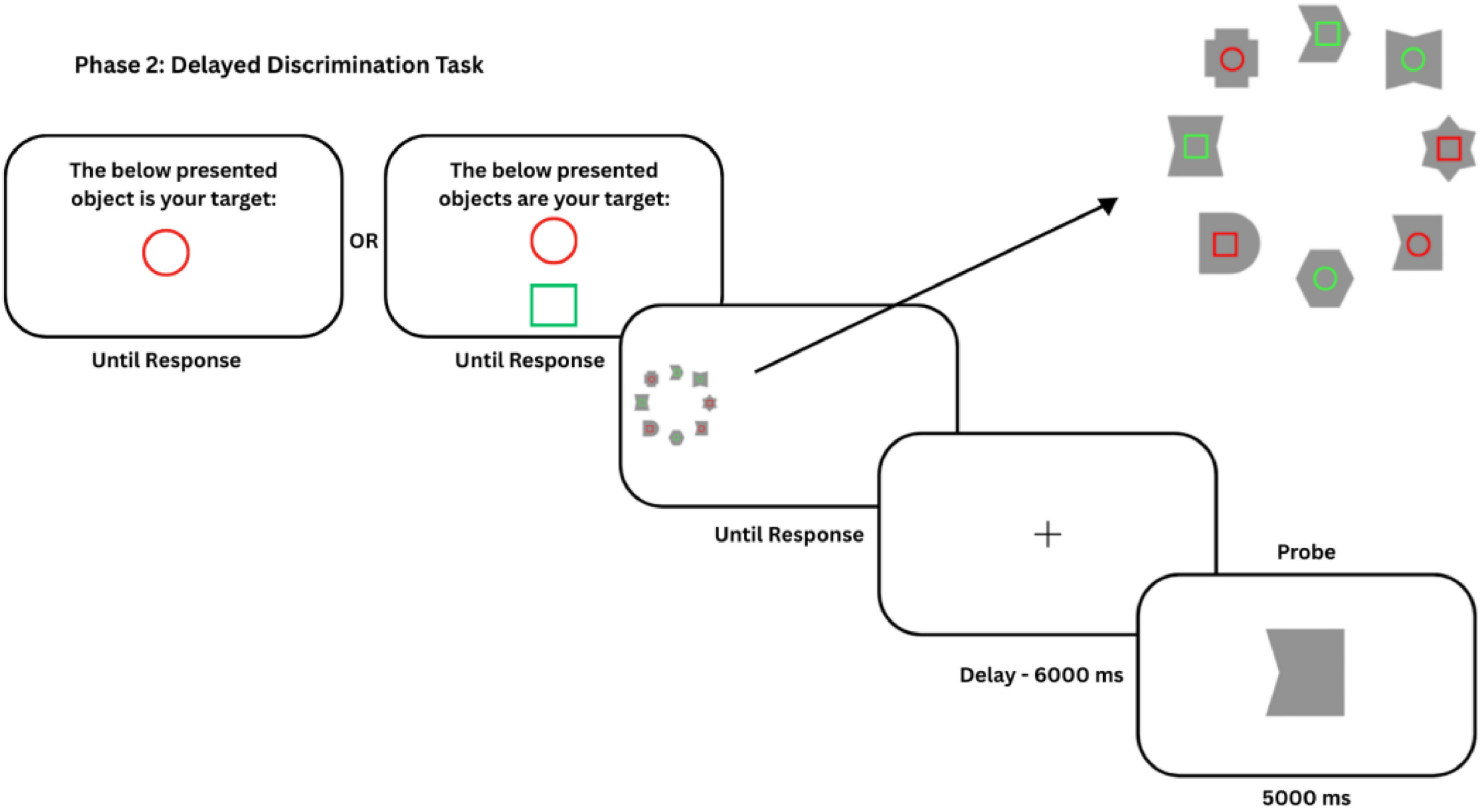
The trial structure for the delayed discrimination task for Experiments 1 and 2. The experiment started with a memory probe that defined the target. There was one target for Experiment 1 (low load) and two targets for Experiment 2 (high load). Each trial started with the memory array. The array of shapes was spatially offset to the left or right side of the screen during the WM task, avoiding overlap with the central fixation area used in Phase 1. Participants had to encode the shapes that contained the target(s). They pressed the space bar after encoding. This was followed by a blank screen window of 6,000 ms. A probe appeared after the delay, prompting a response on whether the target was presented in the probed shape.

This task involved the presentation of complex shapes arranged around the coordinates of an imaginary circle with a visual angle of 5.23° (as in the visual search task; Figure 2). Crucially, unlike the visual search task, the center of this imaginary circle was offset to the (left or right) periphery of the display. That is, the WM object array was presented in the periphery of the screen and not the center. Eight items were sampled from a set of 18 complex shapes.

### Procedure

Each experiment took approximately 50–75 min and consisted of 420 trials total (300 in Phase 1; 120 in Phase 2). Participants completed a 13-trial practice block before beginning the main experiment.

#### Phase 1 - visual search task

In each trial, participants were instructed to ignore the salient color singleton and report the orientation of the line segment inside the odd-shaped target by pressing the “Left” (for horizontal line segment) or “Right” arrow key (for vertical line segment). The search display (Figure 1) remained on screen for 5,000 ms or until a response was made. Incorrect responses or timeouts were signaled by a beep sound. The high-salience distractor appeared in one specific quadrant of the display with high probability (60% of trials), while appearing in the remaining quadrants with lower probability. Each quadrant is a cluster of three adjacent search locations along the imaginary circle (e.g., 12–3 o’clock positions for the top-right quadrant). This high-probability quadrant was randomly assigned to each participant at the beginning of the experiment. After the completion of Phase 1, participants transitioned to Phase 2 after a self-paced break.

#### Phase 2 - delayed discrimination task

At the beginning of phase 2, participants were shown the specific target(s) item to be remembered for the duration of the task (e.g., “Green Circle”). Each trial began with an encoding display where the target item(s) were embedded within the specific complex shapes (Figure 2). In Experiment 1 (Low Load), the target was presented inside two shapes. In Experiment 2 (High Load), there were two targets, and these two targets were embedded in four shapes. Participants were given a duration of their choice to encode the search display and remember the shape in which the target(s) were embedded. Participants pressed the space bar once they encoded the target shapes. A 6,000 ms retention interval followed the keypress, during which participants were presented with a white screen. After this interval, a single probe (drawn from among the shapes presented during encoding) appeared at the center of the screen. Participants indicated whether the probe shape was one of the shapes that had contained a target item in the encoding display (Press “Right arrow key” for Yes, “Left arrow key” for No). The probed shape could be presented in either the HPDL or LPDL (Low-probability Distractor Location) of the visual search task during the encoding phase. The shapes in the HPDL and LPDL locations had an equal probability to be the probe.

## Results

### Experiment 1 (low load)

#### Visual search task

**FIGURE 3:**
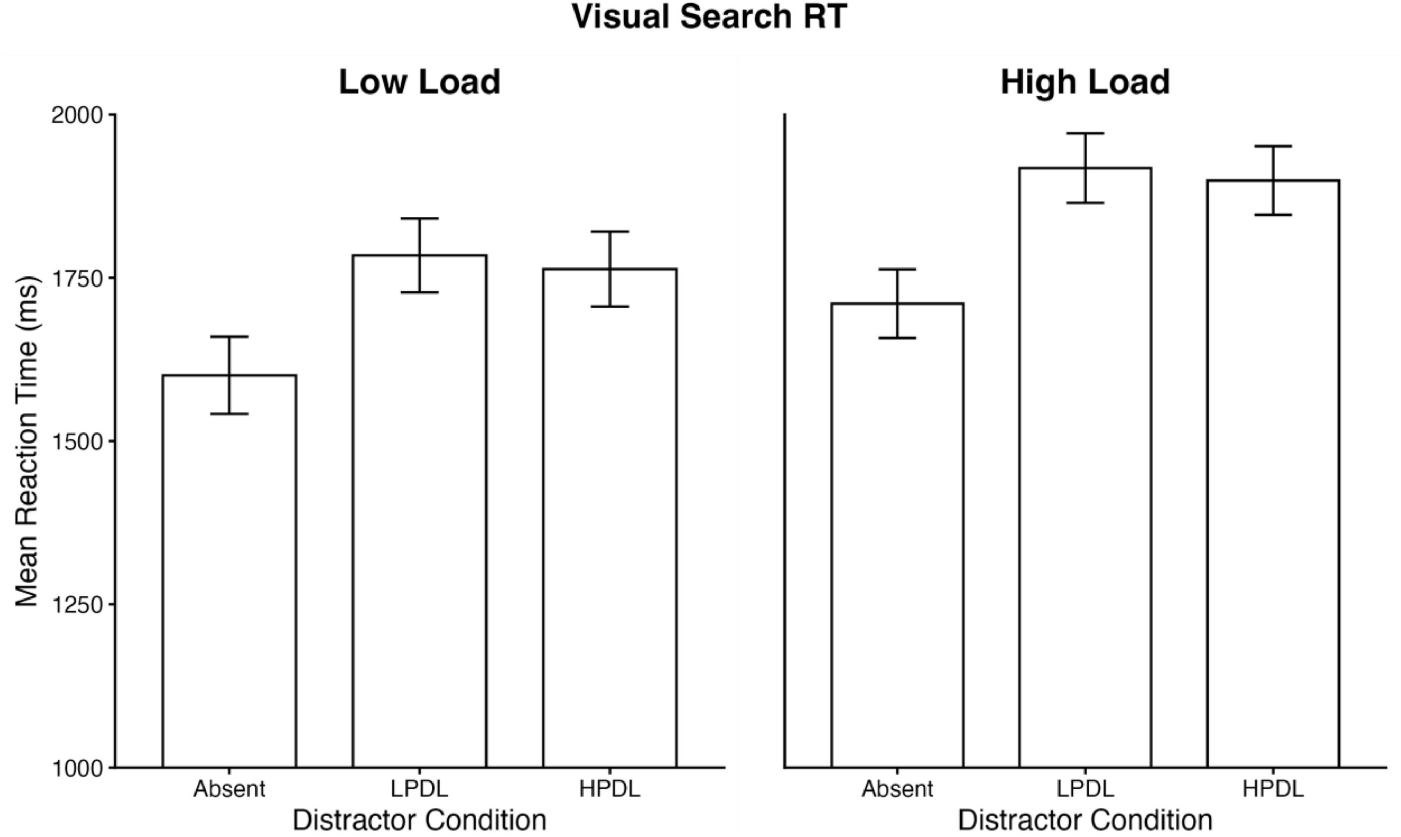
Mean response times (RTs) across conditions in the visual search task for Experiments 1 and 2.

Response times (RTs) faster than 100 ms or slower than 4,000 ms (5.47 %) and incorrect trials were removed from the RT analysis. To examine the effect of distractor probability on search performance, a one-way repeated measures ANOVA was conducted on mean RTs with Distractor Condition (Absent, HPDL, and LPDL) as the within-subject factor. Mauchly’s test indicated a violation of sphericity, and degrees of freedom were corrected using Greenhouse-Geisser estimates (epsilon < 0.75). The analysis revealed a significant main effect of Distractor Condition, F(1.72, 68.74) = 60.13, *p* < 0.001, η_*p*_^2^ = 0.601 (Figure 3). *Post hoc* comparisons using the Tukey adjustment indicated that search times in the Distractor Absent condition were significantly faster than in both the LPDL condition (*t* = 8.88, *p* < 0.001) and the HPDL condition (*t* = 11.41, *p* < 0.001). Critically, however, the comparison between the LPDL and HPDL conditions was not statistically significant [Mean difference = 21 ms, t(40) = 1.08, *p* = 0.531]. ANOVA on Accuracy did not reveal a significant main effect (*p* = 0.52).

#### Delayed discrimination task

Performance in the delayed discrimination task was assessed using Signal Detection Theory (SDT) measures separately for the HPDL and LPDL conditions. Trials were categorized based on the participants’ responses to the probe: a Hit was defined as correctly identifying the presence of target in the probe in the memorized array (Response: “Yes” | Target probed), while a False Alarm (FA) was defined as incorrectly reporting the presence of target at probed shape probe when none appeared (Response: “Yes” | Probe: Absent). To account for extreme values (e.g., hit rates of 1.0 or false alarm rates of 0), a log-linear correction was applied to the raw counts before calculating hit rate (H) and false alarm rate (FA) (Hautus, 1995). Specifically, 0.5 was added to the number of hits and false alarms, and 1 was added to the number of signal and noise trials, respectively. Sensitivity (d’) and response bias (C) were calculated using the formulas:


$$d^{\prime }=z\,\left(H\right)-z\,\left(F\,A\right)$$

C=-0.5⁢[z⁢(H)+z⁢(F⁢A)]

where z represents the inverse of the standard normal cumulative distribution function. A higher d’ indicates greater sensitivity to the probe location, while a positive C value indicates a conservative response bias (tendency to respond “No”) and a negative C value indicates a liberal bias (tendency to respond “Yes”). To determine the effect of distractor repetition on the visual search task in the delayed discrimination task, the sensitivity (d’), criterion, hit rate, and false alarm rate were estimated for each participant. A paired test was conducted to assess the difference between the HPDL and LPDL conditions in the delayed discrimination task.

**FIGURE 4:**
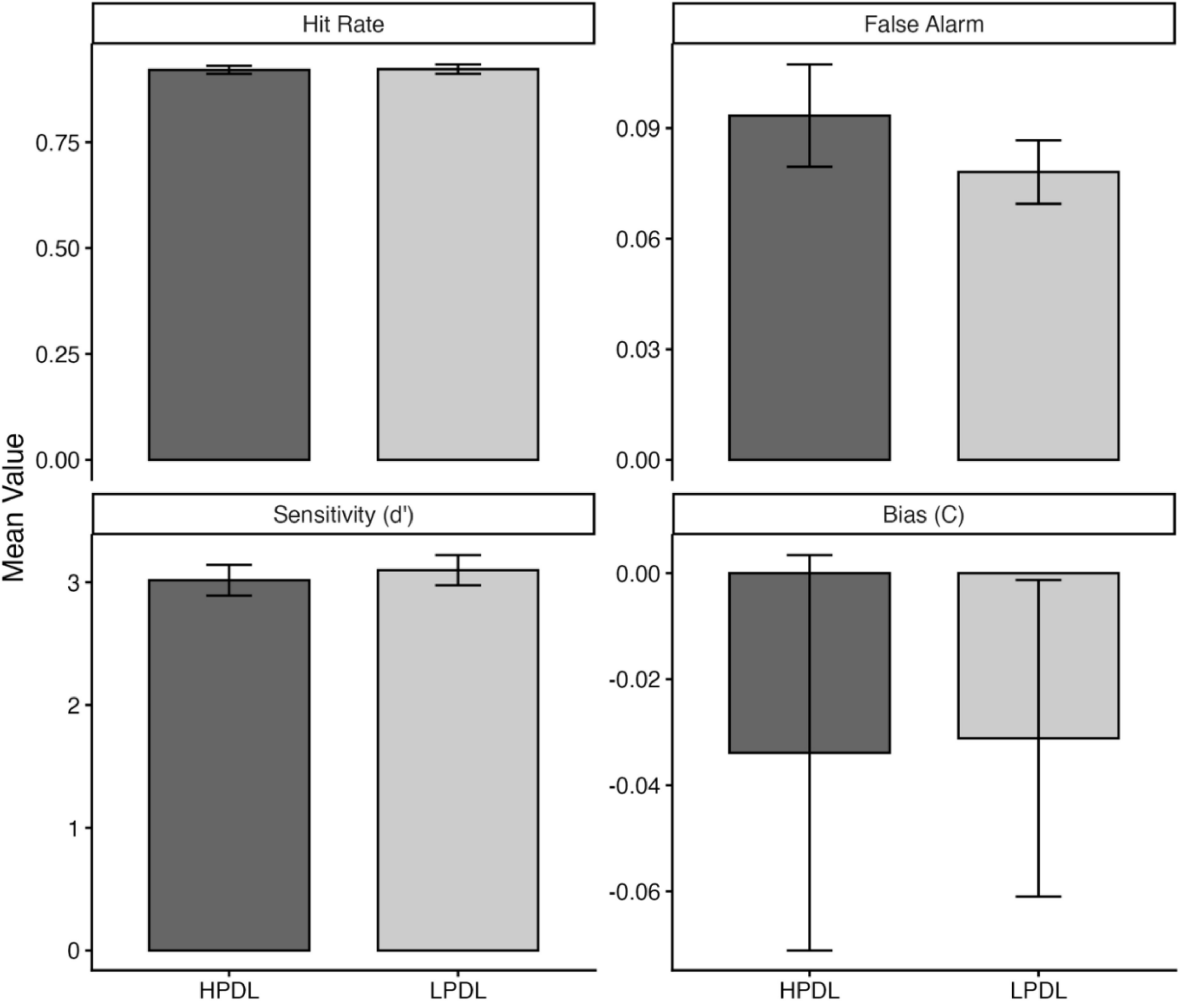
Difference in means of hit rate, false alarm rate, d’, and c for HPDL and LPDL conditions in Experiment 1.

Sensitivity (d’) was not significantly different in trials where the WM target was presented in the HPDL of the visual search task as compared to the LPDL, t(40) = 1.01, *p* = 0.321 (Figure 4). There was no significant difference in the criterion values across HPDL and LPDL conditions, t(40) = 0.06, *p* = 0.954. Further analysis of the hit rate and false alarm rates indicated that participants did not commit significantly more false alarms when the WM target was presented in the HPDL, t(40) = 1.56, *p* = 0.127. There was no significant difference between the hit rates of HPDL and LPDL in the delayed discrimination task, t(40) = 0.21, *p* = 0.833. Together, the analysis of signal detection measures points to a lack of significant difference in WM performance between the HPDL and LPDL conditions, suggesting that under low load, participants did not form strong explicit traces of the distractor locations.

### Experiment 2 (high load)

#### Visual search task

Response times (RTs) faster than 100 ms or slower than 4,000 ms were removed (7.06 %), as were the trials where the participant made an incorrect response, for the visual search RT analysis. A one-way repeated measures ANOVA was conducted on mean RTs with Distractor Condition (Absent, HPDL, and LPDL) as the within-subject factor. The analysis revealed a significant main effect of Distractor Condition, F(1.56, 64.02) = 47.25, *p* < .001, η_*p*_^2^ = 0.54. *Post hoc* comparisons using the Tukey adjustment indicated that search times in the Distractor Absent condition were significantly faster than in both the LPDL condition (t = 7.50, *p* < 0.001) and the HPDL condition (t = 7.47, *p* < .001). However, the comparison between the LPDL and HPDL conditions was not statistically significant (Mean difference = 19 ms, *p* = 0.332). ANOVA on Accuracy did not reveal a significant main effect (*p* = 0.68).

#### Delayed discrimination task

**FIGURE 5:**
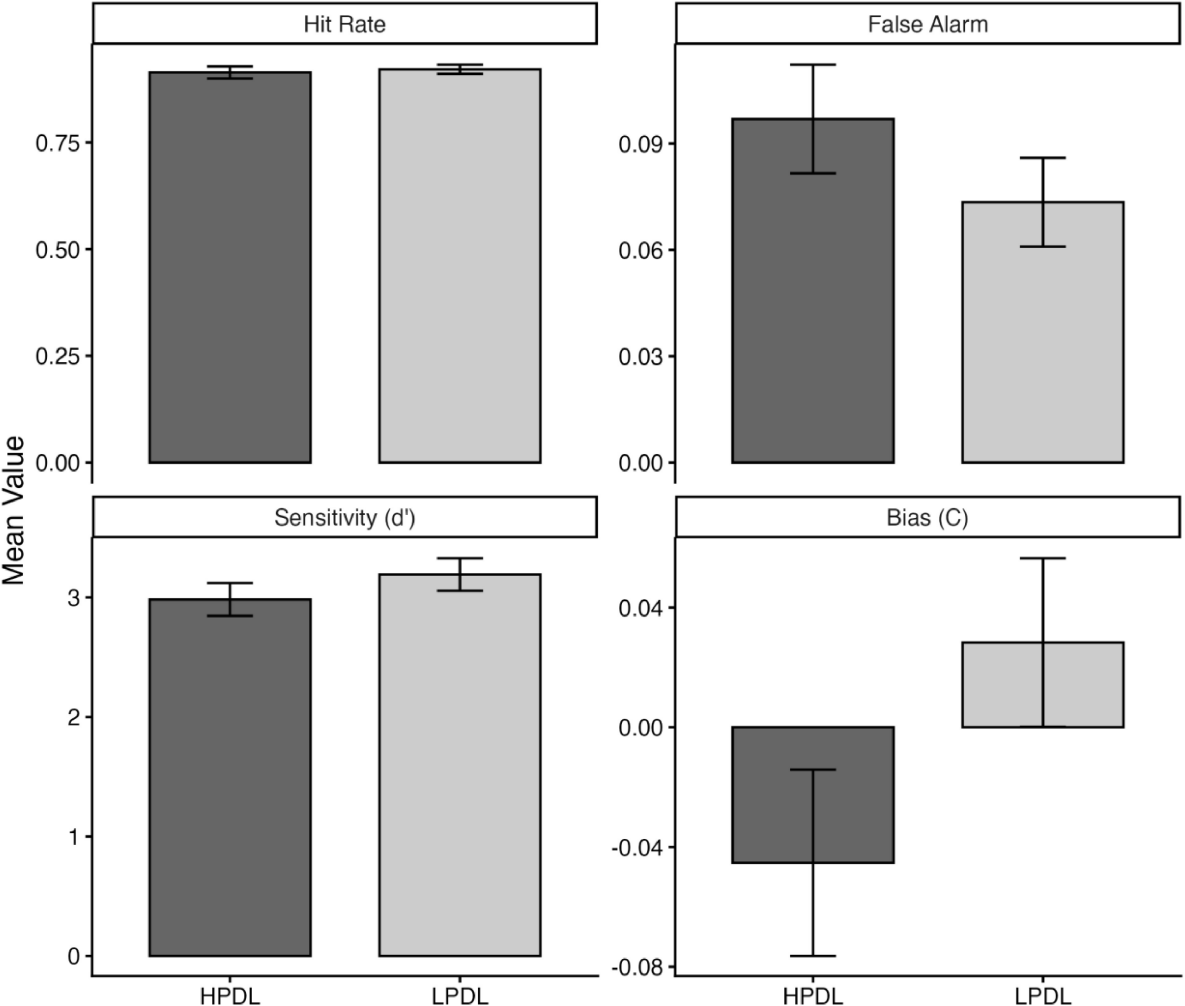
Difference in means of hit rate, false alarm rate, d’, and c for HPDL and LPDL conditions in Experiment 2.

Sensitivity was lower in trials where the WM target was presented in the HPDL of the visual search task as compared to the LPDL, t(42) = 2.03, *p* = 0.04. There was no significant difference in the criterion values across HPDL and LPDL conditions, t(42) = 1.76, *p* = 0.087. While not statistically significant, the numerical difference in criterion values (see Figure 5) suggested a shift toward a more liberal response bias in the HPDL condition compared to the LPDL condition (Figure 5). Further analysis of the hit rate and false alarm rates indicated that participants committed more false alarms when the WM target was presented in the HPDL, t(42) = 2.19, *p* = 0.03. There was no significant difference between the hit rates of HPDL and LPDL in the delayed discrimination task (*p* = 0.43).

Together, the analysis of signal detection measures points to a reduction in sensitivity when the WM target was presented in the HPDL of the visual search task. This difference in performance could not be entirely accounted for by the difference in sensitivity, as the false-alarm rates and criterion values point toward a response bias, where participants made liberal choices when the WM object was presented in HPDL.

### Comparison between Experiment 1 and Experiment 2

To directly test whether the carry-over effect differed significantly between the low-load (Experiment 1) and high-load (Experiment 2) conditions, we conducted a Mixed ANOVA with Experiment (1 vs. 2) as a between-subjects factor and Distractor Condition (HPDL vs. LPDL) as a within-subjects factor. For sensitivity (d’), while the main effect of Distractor Condition was significant across the pooled data, F(1, 82) = 4.83, *p* = 0.03, the interaction between Experiment and Distractor Condition was not significant [F(1, 82) = 0.91, *p* = 0.342]. Similarly, for False Alarm rates, the main effect of Distractor Condition was significant [F(1, 82) = 7.07, *p* = 0.009], but the interaction with Experiment (load) was not significant [F(1, 82) = 0.31, *p* = 0.578].

To quantify the reliability of the effect of load manipulation, we calculated within-subject Cohen’s d (d_z_) for the HPDL vs. LPDL contrast in both experiments. In Experiment 1 (low load), the effect sizes were negligible and not statistically reliable. The effect size for d’ was small (d = −0.10, 95% CI [−0.31, 0.10]), and for false alarms, it was similarly weak (d = 0.18, 95% CI [−0.05, 0.42]), with both confidence intervals crossing zero. In Experiment 2 (high load), the pattern was more reliable. We observed a reliable effect size for d’ (d = −0.23, 95% CI [−0.46, −0.001]) and False Alarm rate (d = 0.25, 95% CI [0.02, 0.48]). This suggests that the effect of distractor regularity on WM performance reached statistical reliability and manifested as a behavioral effect only under high-load conditions.

## Discussion

The present study investigated the carry-over effect of learned distractor suppression on WM representation during encoding and maintenance. Specifically, we examined whether the learned suppression of a high-probability distractor location (HPDL) in a visual search task would have a carry-over effect on a subsequent WM task that required encoding and maintenance of objects at that same location. The carry-over effect of distractor suppression impaired WM representation when targets appeared at the previously suppressed location under high load. These findings provide evidence for a shared spatial priority map where external suppression of environmental distractors fundamentally alters the internal representational quality of WM. However, the effect of this learned suppression on WM was more robust under high load. In Experiment 1, under low WM load, there was no carry-over effect. Performance on the WM task was equivalent for previously suppressed and non-suppressed locations. However, in Experiment 2, under high WM load, a small effect emerged. Participants made significantly more false alarms when the WM target was presented in the previously suppressed location. This led to reduced perceptual sensitivity (d’) and a more liberal response criterion (c) for items of that location.

This carry-over effect of suppression supports the existence of a shared spatial priority map that governs both perceptual selection (external attention) and WM memory (internal attention) (Awh and Jonides, 2001; Klink et al., 2014; Kiyonaga and Egner, 2013; Theeuwes et al., 2022). The sensory recruitment model of WM posits that visual WM is not a separate storage buffer but rather the sustained activation of the same sensory cortical areas used for perception (Postle, 2006; Adam et al., 2022). If statistical learning biases the representations of early visual areas (Ferrante et al., 2018), the learned distractor suppression of HPDL could operate by downregulating the synaptic weights or neural gain at the retinotopic location of the HPDL to filter out distractors. Our results suggest that this down-regulation is persistent and indiscriminate. When the task demands shifted from distractor suppression (visual search) to target encoding and maintenance (delayed discrimination), the visual system could not immediately disengage the suppression. Consequently, the memory targets appearing at the HPDL were processed through a dampened sensory channel, thereby impairing the WM representation. This resulted in a lower signal-to-noise ratio for the encoded traces, leading to the observed reduction in sensitivity. This reduction in sensitivity was driven by a shift toward liberal response bias (Kloosterman et al., 2019; but see Rahnev et al., 2011). Participants committed more false alarms when the WM object was presented at the previously suppressed location.

The emergence of carry-over effects under high load supports the view that selection history and WM are not entirely independent and compete for shared, limited resources. When the WM is taxed, its resources for encoding and maintenance are diminished. In this state, the lingering suppression of a specific spatial location appears to impair the quality of representations at the suppressed location. The specific increase in false alarms suggests that participants had a noisier or less confident representation of items from the suppressed location, leading them to incorrectly identify novel probes as familiar. This aligns with findings by Kuo (2016), who showed that interference from a broader selection history on WM capacity was most pronounced under high-load conditions.

Notably, our results indicate that suppressing a location in the external sensory world results in a corresponding suppression of internal attention and WM at that same coordinate. In Experiment 2, when WM load was high, participants exhibited significantly reduced sensitivity (d’) and increased false alarms for memory targets appearing at the location previously associated with high distractor probability. This occurred even though the delayed discrimination task itself contained no spatial biases, as the WM array was spatially offset from the search array location (center) to the left/right side of the screen, but fell within the same suppression quadrants. This ensured that the impairment in the WM task could not be accounted for by transient habituation but rather points to lasting changes in the attentional priority.

The visual search data did not reveal a significant reduction in RTs for HPDL as compared to LPDL in both experiments, even though there was a marginal reduction in HPDL RTs. This could be because our participants performed 300 trials in the visual search task and the selection history effect takes close to 700 trials to emerge (Wang and Theeuwes, 2018). Although there is insufficient evidence to characterize the effects observed in the WM task as a transfer of suppression learning, the presence of a significant deficit in the WM task suggests that a “latent” spatial bias was indeed acquired. Another caveat in interpreting our results is that the WM task in our study (Mayer et al., 2007) involved both encoding and maintenance. This task cannot dissociate whether the carry-over effects were on the encoding or maintenance stage. Hence, future research could aim to capture the potentially dissociable effects of attentional learning on encoding and maintenance stages of WM, by isolating the effect of encoding time (Vogel et al., 2006) and interference during maintenance (Woodman and Vogel, 2005).

Although the interaction between experiments did not reach significance, the effect size analysis suggests that WM load modulated the latent suppression. The finding that this memory deficit was statistically reliable under high cognitive load (Experiment 2) highlights the resource-dependent nature of attention-WM interaction. We propose that under low load (Experiment 1), available executive control resources (mediated by the prefrontal cortex) can compensate for the degraded sensory input at the HPDL via top-down amplification (Jo et al., 2021). The system could likely effectively “override” the learned suppression when the demand for maintenance is low. However, when WM capacity is taxed (Experiment 2), these executive resources are fully engaged in maintaining the structural integrity of the multiple items (Fougnie and Marois, 2006). The compensatory mechanism fails, and the underlying sensory degradation caused by the learned suppression is unmasked. This pattern strongly favors a *shared resource model* of attention and memory (Ma et al., 2014), contradicting modular accounts that view VSL as a purely automatic, habit-like process independent of WM limits (e.g., Gao and Theeuwes, 2020b). Our data suggest that suppression learning consumes the same finite cognitive resources required for encoding and maintaining memory items, leading to interference when WM load is high.

## Conclusion

In sum, this study demonstrates that spatial suppression acquired through statistical learning can have carry-over effects from a visual search task to a visual WM task, but this effect was reliable when WM load was high. Specifically, the discriminability of WM representation is impaired when the target is presented at a location where the distractor was repeatedly presented (Jiang and Leung, 2005). This suppression of the high-probability distractor location creates carry-over effects on the WM representation and leads to poorer performance, indexed by increased false-alarm rates (Kloosterman et al., 2019). The results suggest a interplay between implicit, history-driven attentional biases and resource-limited executive control.

## Data Availability

The original contributions presented in this study are included in this article/supplementary material, further inquiries can be directed to the corresponding author.
